# A cross-sectional analysis of the predictors of COVID-19 vaccine uptake and vaccine hesitancy in Iraq

**DOI:** 10.1371/journal.pone.0282523

**Published:** 2023-03-09

**Authors:** Saad S. J. Alatrany, Ashraf Muwafa Falaiyah, Raheem Hammlee Maarij Zuhairawi, Ruth Ogden, ‏Hasan ALi Sayyid ALdrraji, Abbas S. S. Alatrany, Dhiya Al-Jumeily

**Affiliations:** 1 Imam Ja’afar Al-Sadiq University, Baghdad, Iraq; 2 College of Education for Human Sciences, Ibn Reshed, University of Baghdad, Baghdad, Iraq; 3 School of Psychology, Liverpool John Moores University, Liverpool, United Kingdom; 4 University of Information Technology and Communications, Baghdad, Iraq; 5 School of Computer Science and Mathematics, Liverpool John Moores University, Liverpool, United Kingdom; Universite Ziane Achour - Djelfa, ALGERIA

## Abstract

**Background:**

Vaccine hesitancy poses a significant risk to global recovery from COVID-19. To date however, there is little research exploring the psychological factors associated with vaccine acceptability and hesitancy in Iraq.

**Aim:**

To explore attitudes towards COVID-19 vaccination in Iraq. To establish the predictors of vaccine uptake and vaccine hesitancy in an Iraqi population.

**Methods:**

Using a cross-sectional design, 7,778 participants completed an online questionnaire exploring their vaccination status, likelihood of infection, perceived severity infection, benefits of vaccine, barriers to vaccine, anticipated regret, subjective norms, and trust in government.

**Findings:**

Vaccination rates increased with age and were greater in males, those who were married, divorced or widowed, those with children and those with underlying conditions. Vaccine hesitancy was widespread with 61.40% of unvaccinated individuals reported an unwillingness to receive a COVID-19 vaccine. In unvaccinated groups, vaccine hesitancy was associated with lower trust in the government, more negative social norms, greater perceived barriers to vaccination and reduced perceived benefits.

**Conclusions:**

There is significant COVID-19 vaccine hesitancy in Iraq. Public health institutions should be aware of the influence of demographic factors, as well as personal beliefs and social norms, on individuals’ decisions to vaccinate. Public health messaging should therefore aim to be tailored to address the concerns of citizens.

## Introduction

The threat to public health resulting from the coronavirus 2019 (COVID-19) global pandemic has led governments around the world to enact mass vaccination programmes aimed at mitigating the negative health outcomes of COVID-19 [1]. As with any mass vaccination programme, the success of this programme is dependent on the extent to which the public are willing to receive vaccinations [2, 3]. Establishing the barriers and facilitators to vaccination is therefore critical to ensuring sufficient vaccine coverage [2, 3].

Vaccine hesitancy describes the refusal to vaccinate, or a delay in the acceptance of vaccination, despite the availability of safe and effective vaccines [3, 4]. Vaccination hesitancy poses a significant threat to global health [5], with the WHO listing vaccination hesitancy among the top ten threats to global health today [3]. This threat is illustrated by several high-profile examples of disease outbreaks following vaccination hesitancy, for example, the 2019 measles outbreak in Samoa following a nursing error [6] and multiple measles outbreaks in the UK and USA due to hesitancy surrounding the MMR vaccine [7–9].

The causes of vaccine hesitancy are complex [2]. Three key factors are thought to be: complacency, convenience and confidence [2]. Complacency refers to the belief that the vaccine is not needed, convenience refers to the extent to which the vaccine is accessible, and confidence refers to trust in the safety and effectiveness of the vaccine [2]. Complacency, convenience and confidence are shaped by a range of factors including disease specific components, public health and media messaging, social norms and trust in the government [10, 11]. They are also affected by demographic factors, with increasing age [12, 13], higher levels of education [14] and male gender [12] all being associated with increased vaccine acceptability and reduced vaccine hesitancy.

Although the COVID-19 vaccines are considered safe and with low risk of severe side effects [15, 16], there is evidence of significant vaccine hesitancy in populations across the world [11, 17, 18]. Whilst there is evidence to suggest that the factors which predict COVID-19 vaccine hesitancy appear to differ across cultures [10], there is a paucity of research exploring COVID-19 vaccine hesitancy in low- and middle-income countries [11]. Understanding the prevalence and culturally specific predictors of COVID-19 vaccine hesitancy in low- and middle-income countries is however critical to ensuring sufficient global vaccine coverage. The current study therefore sought to explore attitudes towards COVID-19 vaccination acceptability in Iraq.

Iraq’s COVID-19 vaccination programme began in May 2021 following the receipt of 336,000 doses of AstraZeneca via COVAX, which were followed by further shipments of Pfizer and Sinopharm COVID-19 vaccines. For the first three months of the vaccination programme, priority for vaccination was given to elderly individuals and those with chronic health conditions (e.g. diabetes) with an overall aim of vaccinating 20% of the population by the end of 2021. However, despite largely achieved the target of vaccinating 20% of the population by the end of 2021, vaccine hesitancy is prevalent in the general population [19, 20] and in health care professionals [21]. There is also evidence that vaccine hesitancy is growing over the course of the pandemic, and thus poses an increasing threat to the efforts to reduce the spread of the virus and its subsequent impact on socialization and economic productivity [22]. It is therefore important to further establish the factors associated with vaccine hesitancy specifically in Iraq.

The current study therefore sought to measure vaccine uptake and vaccine hesitancy in Iraq and establish the demographic and attitudinal predictors of acceptance and hesitancy. A modified version of the vaccine acceptance and hesitancy questionnaire reported in a pre-print of Goodwin et al., (2022) [10] was used to measure vaccine acceptance and hesitancy. In addition, as in Goodwin et al., (2022) [10], the questionnaire also measured factors known to influence vaccine hesitancy, including perceived likelihood of infection, perceived severity of infection, benefits of vaccine, barriers to vaccine, anticipated regret, subjective norms, and trust in government. The questionnaire also recorded gender, marital status, profession, pre-existing chronic diseases, whether an individual had previously been diagnosed with COVID-19 and whether they had experienced the death of a first-degree relative or close friend due to COVID-19. Regression analysis was then used to establish the extent to which these factors were predictive of vaccine acceptance and vaccine hesitancy. The questionnaires were completed between 17^th^ June 2021 and 5^th^ August 2021.

It was expected that vaccine uptake rates would be greater in males than in females and in older than younger individuals, as observed in other countries [12, 13] and because of the vaccination prioritization program in place in Iraq at the time of study. Greater vaccine hesitancy was expected to be associated with increased perceived barriers to vaccination, lower trust in government, lower perceived risks of infection and illness severity and more negative social norms towards vaccination [2, 10–13].

## Methods

### Participants

Seven thousand seven hundred seventy-eight participants were recruited through volunteer sampling. The sample consisted of 4483 (58%) males and 3295 (42%) females. The age of participants ranged from 18 to 68 years (*M* = 42.54, *SD* = 15.49). To take part participants had to be aged 18 years or over and be currently residing in Iraq. Participants were recruited using paid posts on Facebook which contained a link to the questionnaire. The posts were configured to recruited participants from the different cities, genders, and ages in Iraq. The paid post reached 38,678 people, of whom 11,209 clicked on the link in the post (28.90%). Of those who opened the link, 2,569 were under 18 years of age and therefore advised that they could not proceed, and a further 862 either did not agree to participate or did not complete the questionnaire. The discrepancy between the advert being targeted at people over the age of 18 and some participants clicking on the link reporting a younger age reflects the tendency for people of fabricate their ages on social media platforms. The final sample of 7,778 therefore represents 20.11% of whose who saw the advert and 69.39% of those who opened the link. Participants completed the questionnaire between 17^th^ June 2021 and 5^th^ August 2021. Table 1 shows further demographic information as a function of vaccination status. The study was approved by Imam Ja’afar Al-Sadiq University Research Ethics Committee and all participants gave informed written consent by ticking a box on the online form.

**Table 1:** Vaccination rates (%) as a function of demographic groups.

		N	Unvaccinated %	At least 1 vaccine dose %
**Total sample**		**7778**	**32**	**68**
Gender	Male	4482	62	38
	Female	3294	75	25
Marital status	Single	3909	78	22
	Married	3672	57	43
	Divorced	131	62	38
	Widowed	64	58	42
Profession	Student	4096	80	20
	Government Employee	2565	53	47
	Homemaker	158	69	30
	Private Sector	632	59	41
	Job Seeker	121	67	33
	Retired	204	34	66
Has children	Yes	3355	55	45
	No	309	77	23
Chronic Illness	Yes	3674	61	39
	No	1402	69	31
Previous COVID-19 diagnosis	Yes	3315	68	32
	No	4461	67	33
First degree relative diagnosed	Yes	6393	67	33
	No	1382	73	27
Death of first degree relative	Yes	2091	67	33
	No	4299	62	34

### Measures and procedure

The questionnaire was a modified version of the questionnaire published as a pre-print by Goodwin et al., (2022) [10] to establish the psychological factor underpinning vaccine hesitancy. We translated Goodman et al’s (2022) [10] measures of perceived likelihood of infection, perceived severity of illness, perceived benefits to vaccination, perceived barriers to vaccination, trust in Government and social norms into Arabic. We modified Goodwin et al’s (2022) [10] measure of vaccine hesitancy to reflect the fact that Iraq had already commenced its vaccination program and Goodwins original measure was designed for administration prior to the start of a vaccination program. Specifically, we added in a questionnaire asking the participant to report their COVID-19 vaccination status. The questionnaire was delivered online via Google forms. The link to information about the study was included in the Facebook post which advertised the study. Participants who clicked on the link in the advert were then asked to read the study information, confirm they were over 18 years of age and consent to the study. Failure to consent or being under 18 years of age prevented participants from accessing the questionnaire. All questions were presented in Arabic. All participants completed questions exploring demographics and their attitudes towards COVID-19 vaccination. Participants also indicated whether they had received any vaccination for COVID-19 (vaccinated), or whether they were unvaccinated (unvaccinated). Those who were unvaccinated also completed a further series of questions exploring their beliefs about their likelihood of infection with COVID-19, their perceptions of the severity of COVID-19 infection, the perceived benefits of vaccination, the perceived barriers to vaccination, subjective norms, and trust in government. The questionnaire took approximately 11 minutes to complete.

#### Demographic questions

Participants stated their age, gender, marital status, profession and whether they had any children. Participants also indicated whether they suffered from any chronic diseases, whether they had previously been diagnosed with COVID-19, whether a first degree relative has been diagnosed with COVID-19 and whether a first-degree relative had died of from COVID-19.

#### Vaccine hesitancy

Participants were asked to rate their attitude towards COVID-19 vaccination using a 4-point scale in which 1 indicated a desire to be vaccinated (greater vaccine acceptance) and 4 indicated a strong desire to remain unvaccinated (greater vaccine hesitancy).

To assess perceived likelihood of infection, severity of illness, benefits of vaccination, barriers to vaccination and trust in the government, participants rated their level of agreement with the following statements using a 5-point likert scale ranging from 1. Strongly disagree to 5, Strongly agree. For each sub-scale, responses were added together to give a total score for that sub-scale.

### Perceived likelihood of infection

1. My chance of getting COVID-19 in the next few months is great.
2. I am worried about the likelihood of getting covid-19 in the near future.
3. Getting COVID-19 is a current possibility for me.

A higher score on this subscale indicated greater belief in the likelihood of infection. Cronbach’s alpha for this sub-scale was α = .82.

### Perceived severity of illness

1. Complications from COVID-19 are severe.
2. I will be very sick if I get COVID-19
3. I am afraid of getting COVID-19.

A higher score on this subscale indicated greater belief that covid-19 is a severe illness. Cronbach’s alpha for this sub-scale was α = .80.

### Perceived benefits to vaccination

1. Vaccination is a good idea because I will feel less worried about catching COVID-19
2. Vaccination decreases my chance of getting COVID-19 or its complications.
3. If I get vaccinated I will decrease the risk of spreading the disease to others.

A higher score on this subscale indicated greater perceived benefits to vaccination. Cronbach’s alpha for this sub-scale was α = .91.

### Perceived barriers to vaccination

1. The side effects of COVID-19 will interfere with my usual activities.
2. I cannot be bothered to get a COVID-19 vaccination.

A higher score on this subscale indicated greater perceived barriers to vaccination. Cronbach’s alpha was for this sub-scale was α = .45.

#### Trust in the government

To assess trust in the government, participants rated their agreement with the following three statements using a 5-point likert scale in which 1 represented agree and 5 represented strongly disagree.

1. I trust the Government in general.
2. I trust the Government to deal with COVID-19.
3. I trust the Government regarding vaccination.

A higher score on this subscale indicated higher level of distrust in government. Cronbach’s alpha for this sub-scale was α = .92.

#### Social norms

To assess social norms, participants rated their agreement with the following seven statements using a 7-point likert scale in which 1 represented strongly disagree and 7 represented strongly agree.

1. People who are important to me would approve of me having the COVID-19 vaccination.
2. My family would approve of me having the COVID-19 vaccination.
3. My friends would approve of me having the COVID-19 vaccination.
4. I feel under pressure to have a COVID-19 vaccination.
5. People who are important to me influence my decision to have the COVID-19 vaccination.

A higher score on this subscale indicated more positive subjective norms for vaccination. Cronbach’s alpha for this sub-scale was α = .84.

### Data analysis

A participant was deemed to have accepted a COVID-19 vaccine if they had received one or more dose of any COVID-19 vaccination. The effects of demographic factors (gender, marital status, child-status, profession, chronic illness status, previous diagnosis and whether a relative had previously had COVID-19 or died from COVID-19) on vaccination uptake rates were analyzed using chi-square. Post-hoc tests were conducted on the chi-square analysis using adjusted residuals which were corrected for multiple comparisons. This analysis was followed up logistical regression analysis to determine the factors which were predictive of vaccine uptake.

Vaccine hesitancy was only assessed in unvaccinated respondents. The factors associated with vaccine hesitancy were analyzed using ordinal regression. Predictor variables included demographic factors, perceived likelihood of infection, perceived severity of infection, benefits of vaccine, barriers to vaccine, anticipated regret, subjective norms, and trust in government.

## Results

### Vaccine uptake rates

Chi-square analysis was conducted to assess the association between vaccine status (vaccinated vs unvaccinated) and the demographic factors listed in Table 1. There was a significant association between gender and vaccination status *X*^*2*^ (1) = 156.22, *p* < .001 with males showing greater vaccination rates than expected by chance and females showing lower than expected vaccination rates (*p* < .001). There was a significant association between marital status and vaccination status *X*^*2*^ (3) = 351.51, *p* < .001 with greater vaccination rates in married people than expected by chance (*p* < .001) and fewer in single people than expected by chance (*p* < .001). There was a significant association between having children and vaccination status *X*^*2*^ (1) = 88.80, *p* < .001 with greater than expected vaccination rates in those with children (*p* < .001) and lower than expected vaccination rates in those without children (*p* < .001). There was a significant association between profession and vaccination status *X*^*2*^ (5) = 650.42, *p* < .001 with significantly lower vaccination rates in students than would be expected by chance (*p* < .001) and significantly greater vaccination rates in government employees (*p* < .001), private sector employees (*p* < .001) and the retired (*p* < .001). There was a significant association between chronic illness and vaccination status *X*^*2*^ (1) = 34.17, *p* < .001 with individuals with chronic illness showing greater vaccination rates than expected (*p* < .001) and individuals without chronic illness having lower than expected vaccination rates (*p* < .001). There was also a significant association between having a first degree relative diagnosed with COVID-19 and vaccination status *X*^*2*^ (1) = .20.31, *p* < .001, with those with relatives who have had COVID-19 more likely to have been vaccinated than expected (*p* < .001) and those without being less likely to have been vaccinated (*p* < .001). There was no significant association between having previously been diagnosed with COVID-19 and vaccination status *X*^*2*^ (1) = .25, *p* = .62. Nor was there a significant association between having a relative who has died from COVID-19 and vaccination status *X*^*2*^ (1) = .29, *p* = .59.

A logistic regression was performed to ascertain the effects of age, gender, marital status, child status, profession, chronic disease status and having a relative diagnosed with COVID-19 on the likelihood of vaccination. The model was statistically significant, χ2(13) = 394.26, *p* < .001. The model explained 15.00% (Nagelkerke R^2^) of the variance in vaccination status and correctly classified 64.30% of cases. Age was a significant predictor of vaccination status with greater age associated with an increased likelihood of being vaccinated (Wald = 76.27, *p* < .001, odds ratio of 1.04, 95% CI 1.03–1.04). Gender was a significant predictor of vaccination status (Wald = 50.00, p < .001) with males being .56 times more likely to be vaccinated than females (95% CI .47 - .66). Child status was a significant predictor of vaccination status (Wald = 15.86, *p* < .001), with people with children 1.62 times more likely to be vaccinated than those without children (95% CI 1.27–2.06). Having a chronic disease was a significant predictor of vaccination status (Wald = 8.56, *p* = .003), with people with chronic disease were .78 times more likely to be vaccinated than those without chronic disease (95% CI .67 –.92). Being previously diagnosed with COVID-19 was also a significant predictor of vaccination status (Wald = 14.65, *p*, .001), with people with a previous diagnosis of COVID-19 being .76 times less likely to be vaccinated. Although profession was a significant predictor of vaccination status (Wald = 37.91, *p* < .001), being a student was the only significant predictive category with students being .45 (95% CI .30 - .69) times less likely to be vaccinated than other groups. Marital status (Wald = 5.80, p = .06) and having a first degree relative diagnosed with COVID-19 (Wald = 3.72, p =. 06) were not significant predictors of vaccination status.

### Analysis of people who were unvaccinated

**Fig 1:**
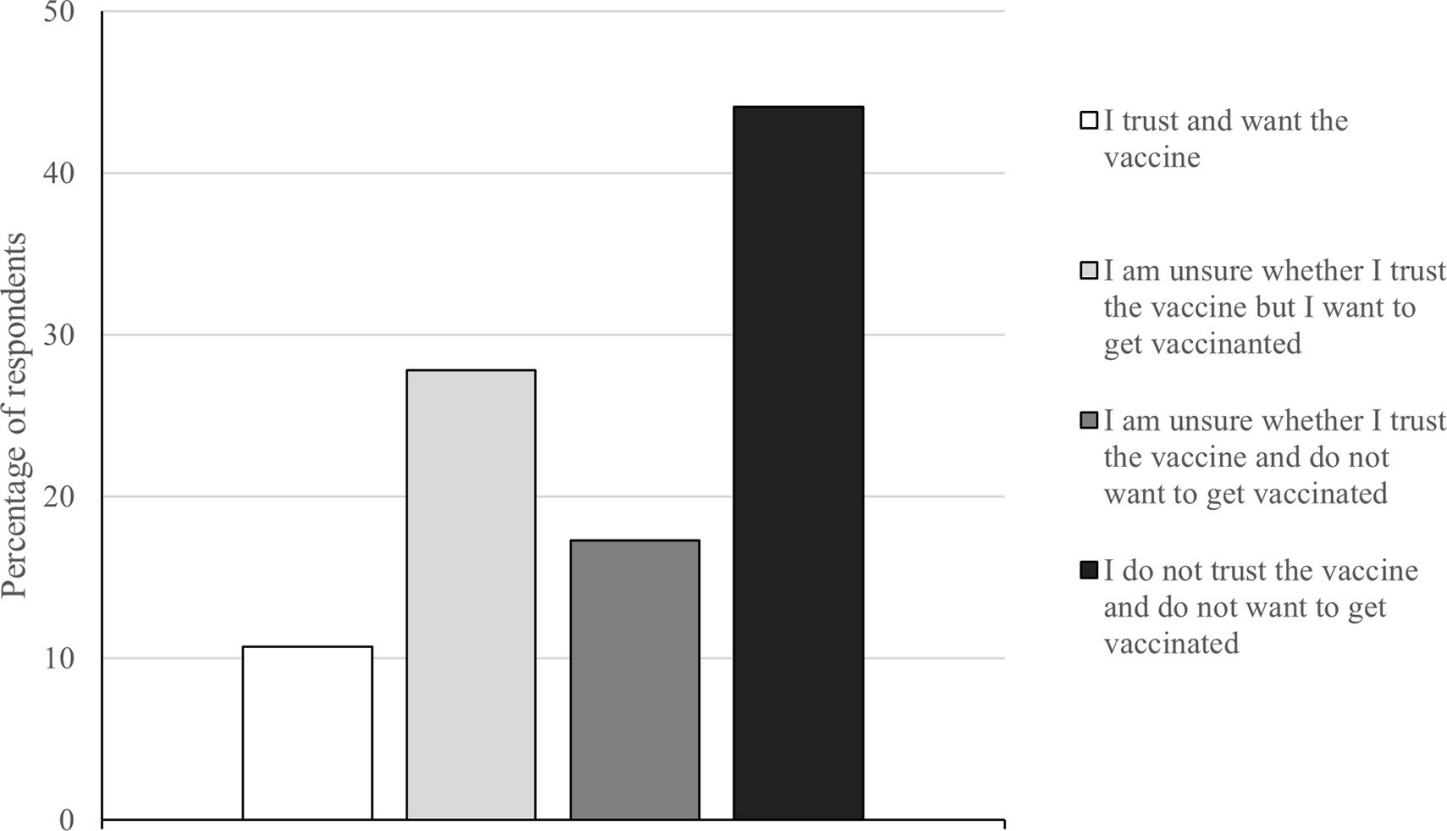
Response frequencies of unvaccinated participants attitudes towards future covid-19 vaccination.

Participants who were unvaccinated rated their attitude towards vaccination using a 4-point scale in which a higher score indicates a more negative attitude towards vaccination. They also rated their trust in the government, social norms, perceived benefits and barriers to vaccination and perceived illness severity. Self-reported levels of vaccine hesitancy are displayed in Fig 1.

Examination of Fig 1 suggest widespread vaccine hesitancy with 61.40% of participants reporting an unwillingness to receive a COVID-19 vaccination in the future.

Ordinal regression with proportional odds was conducted to establish the effect of demographic factors, trust in the government, social norms, perceived likelihood of infection, perceived benefits and barriers to vaccination and perceived severity of COVID-19 on attitudes towards future vaccination. The model was a statistically significant, χ^2^(19) = 1094.20 *p* < .001 fit for the data, with pseudo R squared values of .20 - .42. Trust in the government (Wald = 43.86, *p* < .001, odds ratio .92, 95% CI .82 –.94), social norms (Wald = 15.45, *p* < .001, odds ratio .98, 95% CI .97 - .99), perceived benefits of vaccination (Wald = 437.50, *p* < .001, odds ratio .72 95% CI .70 –.75), perceived severity of COVID-19 (Wald = 10.52, *p* = .001, odds ratio 1.05 95% CI 1.02–1.08), perceived barriers to vaccination (Wald = 9.24, *p* = .002, odds ratio 1.07, 95% CI 1.02–1.11) and not having a relative having had COVID-19 (Wald = 6.91, *p* = .009, odds ratio 1.40, 95% CI 1.09–1.80) were all significant predictors of vaccine hesitancy. Perceived infection likelihood (*p* = .56), age (*p* = .59), gender (*p* = .79), marital status (*p* = .78), child status (*p* = .82), chronic disease status (*p* = .20), previous COVID-19 diagnosis (*p* = .89) and profession (*p* = .10-.90) were not significant predictors of attitudes to vaccination. Greater vaccine hesitancy was therefore associated with less trust in the government, less positive social norms, fewer perceived benefits to vaccination, greater perceived illness severity, greater perceived barriers to vaccination and not having a relative with COVID-19.

## Discussion

This study aimed to explore the factors affecting COVID-19 vaccine uptake and vaccine hesitancy in Iraq. The findings show that, at the time of study, a range of demographic and attitudinal factors influenced vaccine uptake and vaccine hesitancy.

### Vaccine uptake

Of the 7,778 participants surveyed, 32.40% had received one or more dose of a COVID-19 vaccine. This was higher than the general level of vaccination in Iraq at the time of study (17.7%) suggesting greater propensity for vaccination in our sample than the general population. Analysis of vaccine uptake rates suggests that rates of vaccination varied across different demographic groups. Significant predictors of vaccine acceptance were increasing age and being male, having children, having no previous diagnosis of COVID-19, having a chronic illness and not being a student. Although vaccination rates were greater in those who had a relative who had COVID-19, it was not a predictive factor of vaccination. Marital status also had no influence on vaccination rates.

Greater levels of vaccination in male than female Iraqis confirms previous reports of greater male vaccination uptake in Iraq [22], other low-, middle- and some high-income countries [10–13]. Whilst there therefore appears to be *some* cross-cultural universality in the effect of gender on vaccination rates, it is notable that there is some evidence of greater levels of vaccine up-take in females than in males in the USA [23]. Gender inequality in the uptake of vaccination poses a significant threat to public, and in particular maternal health [24]. Pregnant women are at increased risk of serious complications from COVID-19 (e.g. invasive ventilation and intense care treatment) and are more likely to deliver pre-term babies requiring specialist care [24]. In Iraq, the combination of low vaccine uptake in women, enhanced vulnerability of pregnant women to COVID-19, and limited access to specialist obstetric care [25] may significantly impact maternal mortality. There is therefore a need for research to establish the barriers and facilitators of vaccination acceptance in women of childbearing age in Iraq, and targeted public health messaging aimed at these groups.

Greater vaccination rates in older than younger individuals, and in those with chronic diseases than those without may indicate greater levels of vaccine acceptance in these groups. Indeed, there is some evidence that similar associations have been observed in other countries [12, 13]. However, it is also possible that this finding is an artefact of the Iraq’s vaccination delivery strategy because during the first three months of the vaccination programme, priority was given to elderly individuals and those with chronic health conditions (e.g. diabetes). The negative effect of previous infection on vaccine uptake rates contradicts previous suggests from studies conducted in the Europe, the USA and Japan and Hong Kong [26] which suggested that prior infection neither increases nor decreases the likelihood of subsequent vaccination.

### Vaccine hesitancy

Analysis of unvaccinated people’s attitudes towards future covid-19 vaccination revealed substantial vaccine hesitancy. Of the unvaccinated respondents surveyed, 61.40% report an unwillingness to obtain a covid-19 vaccination in the future and only 38.60% reported a willingness to receive a covid-19 vaccination in the future. This figure is comparable with that reported by CARE in Iraq in July 2021 who observed that 68% of respondents that they surveyed would be unwilling to have a COVID-19 vaccination [27]. Comparison of vaccine hesitancy rates in Iraq with those in other lower- and middle-income countries suggests that rates of vaccine hesitancy are greater in Iraq than elsewhere. In a review of vaccine hesitancy in low- and middle-income countries, Solis Arce et al., (2021), [28] reported that an average of 80.30% of participants low- and middle-income countries reported a willingness to receive a COVID-19 vaccine, this contrasts with 38.60% in the current study. Whilst this may indicate that vaccine hesitancy poses a greater threat to public health in Iraq than other low- and middle-income countries, it is important to note that the attitudes reported may not translate into comparable rates of vaccine uptake and refusal [29]. Nonetheless, the reported levels of vaccine hesitancy highlight the urgent need for public health messaging aimed to increase vaccine acceptability.

Analysis of the factors associated with vaccine hesitancy revealed that greater vaccination hesitancy was associated with less positive social norms, fewer perceived benefits and more perceived barriers to vaccination, greater perceived severity of illness, lower trust in Government and a lack of family experience of relatives with COVID-19. Interestingly, demographic factors such as age, gender, chronic disease status and number of children did not appear to be associated with vaccination hesitancy.

The relationships between social norms, perceived benefits and barriers to vaccination and trust in Government replicate findings from other territories, including low- and middle-income [28] and high-income countries [8]. The importance of these factors in vaccine hesitancy in Iraq suggests that the three C’s of vaccine hesitancy; convenience, complacency and confidence [2] largely extend to Iraq. Reduced convenience being associated with increased hesitancy is demonstrated by the association between barriers to vaccination and hesitancy. Increased complacency being associated with increased hesitancy is evidenced by the association between social norms and increased hesitancy. Finally, the role of confidence is evidenced by the association between low trust in government and greater vaccine hesitancy. Interestingly however, contrary to expectations, greater vaccine hesitancy was associated with greater perceived illness severity, thus highlighting that complacency surrounding vaccination does not appear to be solely due a lack of concern over COVID-19 itself.

The role of individual, broader societal factors (social norms) and political (trust in Government) factors in vaccine hesitancy in Iraq reinforces the need for clear public health messaging which addresses citizen’s concerns in relation to these factors. Public knowledge about COVID-19 is generally considered to be high in Iraq [30, 31]. However, like many other nations, Iraq has seen increases in the use of social media to share inaccurate information about COVID-19 [32]. Combating this type of misinformation may therefore be critical to improving trust and promoting vaccination as a social norm. Furthermore, the use of trusted and respected public figures to promote vaccination may also assist in increasing trust and social norms.

Unlike observations in many other countries [11–13, 28], there was no effect of age or gender on vaccination hesitancy in Iraq. This highlights the importance of establishing the predictors of vaccine hesitancy across cultures to ensure that public health messaging accurately targets the critical local drivers of hesitancy. Interestingly, in the current study, despite there being no effect of gender on vaccination hesitancy, females were significantly less likely to have a received a COVID-19 vaccine than males. This perhaps implies that the relatively lower vaccination rates in women are due to gender specific barriers that females in Iraq face when accessing vaccinations, rather than greater vaccine hesitancy in this group per se. For example, greater levels of unpaid carer burden in women than men [33, 34], may limit females’ opportunities for vaccination. Furthermore, women’s reduced power and autonomy in household decision making ability [35] may limit women’s access to vaccination facilities. This latter issue highlights the need for campaigns aimed at men to highlight the importance of vaccination for women and girls in their family and community. Clinicians should therefore consider explaining the importance of female vaccination to men who attend for their own vaccinations.

### Limitations

The current study employed an opportunity sampling method. As a result, the sample is not representative of the population. In particular, the use of social media for recruitment limited numbers of participants were obtained from groups who do not typically use social media i.e. older individuals. Similarly, the use of an online form to collect questionnaire responses prevented those without access to the internet from participating. We are therefore limited in the conclusions that we can draw about these groups of individuals. Furthermore, the study design prevented the same participants from responding to the questionnaire raising the possibility that some responses may be duplicates. However, given that participants were not rewarded for participating, we believe the likelihood of this happening is low. Future studies should therefore seek to recruit stratified and representative samples from across Iraq and prevent the possibility of multiple responses.

Although our analysis of vaccine uptake rates indicates that a number of demographic factors were predictive of vaccine uptake rates, it is important to note that, for the first three months of the vaccination program in Iraq, priority was given to elderly individuals and those with chronic health conditions (e.g. diabetes). During this three-month period, these program priorities will have limited vaccine access for younger, well individuals in addition to the predictors observed in this study. It is therefore important to re-assess vaccine hesitancy in Iraq during a period in which all citizens have equal access to vaccination.

This study used a modified version of the vaccine hesitancy measure developed in Goodwin et al. (2022) [10]. Our analysis of the reliability of the subscales in this measure indicated that all but the ‘perceived barriers to vaccination’ subscale had good to excellent internal consistency. The low level of internal consistency for the perceived barriers to vaccination subscale suggests that this element may require refinement. One particular area for future consideration is the extent to which this item explores actual barriers to vaccination. Goodwin et al., (2022) [10] developed the measure for use prior to the commencement of a vaccination program. The low consistency we observed may therefore reflect the use of a measure designed for implementation in a period in which vaccines were not available. A further consequence of this is that current study did not explore actual barriers to vaccination, just perceived ones. Furthermore, the study did not analyse the potential effect of socio-economic status on vaccine uptake and hesitancy. Given the high levels of vaccine hesitancy observed, it is essential that research is conducted to understand the key local factors which promote perceptions of barriers to vaccinations.

Finally, this data was collected in 2021 and may not therefore changes in attitudes to COVID-19 vaccination that may have resulted overtime. Nonetheless, the data provides a record of attitudes towards COVID-19 vaccination at the time of data collection and will therefore serve as a benchmark for changes in attitudes overtime.

## Conclusions

The results of this study suggest that whilst vaccination uptake in our sample exceeded the target set by the Iraqi Government at the time of study, there was significant vaccination hesitancy among unvaccinated individuals. Over 60% of the unvaccinated people surveyed reported an unwillingness to obtain a COVID-19 vaccination in the future. Both vaccine acceptance and vaccine hesitancy were predicted by a range of demographic, individual, social and political factors. In particular, greater vaccine hesitancy was associated with less positive social norms, fewer perceived benefits and more perceived barriers to vaccination, lower trust in government and a lack of family experience of relatives with COVID-19. To improve vaccine uptake, targeted public health messaging should focus addressing citizens’ concerns in these areas, and, in particular in relation to female vaccination.

## Supporting information

S1 Data (SAV)

## References

[1] Le TT, Andreadakis Z, Kumar A, Román RG, Tollefsen S, Saville M, Mayhew S. The COVID-19 vaccine development landscape. Nat Rev Drug Discov. 2020 May 1;19(5):305–6. doi: 10.1038/d41573-020-00073-5 32273591

[2] MacDonald NE. Vaccine hesitancy: Definition, scope and determinants. Vaccine. 2015 Aug 14;33(34):4161–4. doi: 10.1016/j.vaccine.2015.04.036 25896383

[3] World Health Organization (WHO). Ten Threats to Global Health in 2019. Available online: https://www.who.int/news-room/spotlight/ten-threats-to-global-health-in-2019 [accessed 20 September 2021].

[4] Majid U, Ahmad M. The factors that promote vaccine hesitancy, rejection, or delay in parents. Qualitative Health Research. 2020 Sep;30(11):1762–76. doi: 10.1177/1049732320933863 32597313

[5] de Swart R, Takeda M. Editorial overview: combating measles during a COVID-19 pandemic. Current Opinion in Virology. 2020 Apr 1;41:iii–vii. doi: 10.1016/j.coviro.2020.08.008 32919592

[6] Craig AT, Heywood AE, Worth H. Measles epidemic in Samoa and other Pacific islands. The Lancet Infectious Diseases. 2020 Mar 1;20(3):273–5. doi: 10.1016/S1473-3099(20)30053-0 32112752

[7] Cooper LZ, Larson HJ, Katz SL. Protecting public trust in immunization. Pediatrics. 2008 Jul;122(1):149–53. doi: 10.1542/peds.2008-0987 18595998

[8] Larson HJ, Clarke RM, Jarrett C, Eckersberger E, Levine Z, Schulz WS, et al. Measuring trust in vaccination: A systematic review. Human vaccines & immunotherapeutics. 2018 Jul 3;14(7):1599–609. doi: 10.1080/21645515.2018.1459252 29617183 PMC6067893

[9] Lo NC, Hotez PJ. Public health and economic consequences of vaccine hesitancy for measles in the United States. JAMA pediatrics. 2017 Sep 1;171(9):887–92. doi: 10.1001/jamapediatrics.2017.1695 28738137 PMC5710408

[10] Goodwin R, Ben-Ezra M, Takahashi M, Luu LA, Borsfay K, Kovács M, et al. Psychological factors underpinning vaccine willingness in Israel, Japan and Hungary. Scientific reports. 2022 Jan 10;12(1):1–9. 35013430 10.1038/s41598-021-03986-2 PMC8748514

[11] Sallam M. COVID-19 vaccine hesitancy worldwide: a concise systematic review of vaccine acceptance rates. Vaccines. 2021 Feb 16;9(2):160. doi: 10.3390/vaccines9020160 33669441 PMC7920465

[12] Murphy J, Vallières F, Bentall RP, Shevlin M, McBride O, Hartman TK, et al. Psychological characteristics associated with COVID-19 vaccine hesitancy and resistance in Ireland and the United Kingdom. Nature communications. 2021 Jan 4;12(1):1–5. 10.1038/s41467-020-20226-9 PMC7782692 33397962

[13] Sherman SM, Smith LE, Sim J, Amlôt R, Cutts M, Dasch H, et al. COVID-19 vaccination intention in the UK: results from the COVID-19 vaccination acceptability study (CoVAccS), a nationally representative cross-sectional survey. Human vaccines & immunotherapeutics. 2021 Jun 3;17(6):1612–21. 33242386 10.1080/21645515.2020.1846397 PMC8115754

[14] Myers LB, Goodwin R. Using a theoretical framework to determine adults’ intention to vaccinate against pandemic swine flu in priority groups in the UK. Public Health. 2012 Sep 1;126:S53–6. doi: 10.1016/j.puhe.2012.05.024 22784583

[15] World Health Organization. Coronavirus disease (COVID-19): vaccines safety. 2021. Available at https://www.who.int/news-room/q-a-detail/coronavirus-disease-(covid-19)-vaccines-safety [Accessed 23 June 2021].

[16] CDC. Ensuring COVID-19 vaccine safety in the US. 2021. Available at https://www.cdc.gov/coronavirus/2019-ncov/vaccines/safety.html [Accessed January 27 2022].

[17] Lane S, MacDonald NE, Marti M, Dumolard L. Vaccine hesitancy around the globe: Analysis of three years of WHO/UNICEF Joint Reporting Form data-2015–2017. Vaccine. 2018 Jun 18;36(26):3861–7. doi: 10.1016/j.vaccine.2018.03.063 29605516 PMC5999354

[18] Wagner AL, Masters NB, Domek GJ, Mathew JL, Sun X, Asturias EJ, et al. Comparisons of vaccine hesitancy across five low-and middle-income countries. Vaccines. 2019 Oct 18;7(4):155. doi: 10.3390/vaccines7040155 31635270 PMC6963484

[19] Abdulah DM. Prevalence and correlates of COVID‐19 vaccine hesitancy in the general public in Iraqi Kurdistan: A cross‐sectional study. Journal of medical virology. 2021 Dec;93(12):6722–31. doi: 10.1002/jmv.27255 34347294 PMC8427006

[20] Tahir AI, Ramadhan DS, Piro SS, Abdullah RY, Taha AA, Radha RH. COVID-19 vaccine acceptance, hesitancy and refusal among Iraqi Kurdish population. International Journal of Health Sciences. 2022 Jan;16(1):10. 35024029 PMC8721214

[21] Luma AH, Haveen AH, Faiq BB, Stefania M, Leonardo EG. Hesitancy towards Covid-19 vaccination among the healthcare workers in Iraqi Kurdistan. Public Health in Practice. 2022 Jun 1;3:100222. doi: 10.1016/j.puhip.2021.100222 34909704 PMC8662956

[22] Krah K., & Phadera L. (2022). COVID-19 Vaccine Inequities and Hesitancy in Iraq: Results from June, July, and August 2021 Rounds of Iraq High Frequency Phone Survey (IHFPS).

[23] The Gender Vaccine Gap: More Women Than Men Are Getting Covid Shots Available from https://khn.org/news/article/gender-vaccine-gap-more-women-than-men-vaccinated-against-covid/ [accessed 16.01.2023].

[24] Allotey J, Stallings E, Bonet M, Yap M, Chatterjee S, Kew T, et al. Clinical manifestations, risk factors, and maternal and perinatal outcomes of coronavirus disease 2019 in pregnancy: living systematic review and meta-analysis. bmj. 2020 Sep 1;370. doi: 10.1136/bmj.m3320 32873575 PMC7459193

[25] WHO EMRO in Iraq: Maternal, newborn, child and adolescent health: Available from http://www.emro.who.int/pdf/iraq/priority-areas/maternal-newborn-child-and-adolescent-health.pdf?ua=1 [Accessed 28.08.2022].

[26] Troiano G, Nardi A. Vaccine hesitancy in the era of COVID-19. Public health. 2021 May 1;194:245–51. doi: 10.1016/j.puhe.2021.02.025 33965796 PMC7931735

[27] CARE: COVID-19 Vaccination Uptake. Available from https://www.care.at/wp-content/uploads/2021/08/Iraq-Vaccination-Uptake-Study-CARE_FINAL.pdf [Accessed on 27.07.2022].

[28] Solís Arce JS, Warren SS, Meriggi NF, Scacco A, McMurry N, Voors M, et al. COVID-19 vaccine acceptance and hesitancy in low-and middle-income countries. Nature medicine. 2021 Aug;27(8):1385–94. doi: 10.1038/s41591-021-01454-y 34272499 PMC8363502

[29] McEachan RR, Conner M, Taylor NJ, Lawton RJ. Prospective prediction of health-related behaviours with the theory of planned behaviour: A meta-analysis. Health psychology review. 2011 Sep 1;5(2):97–144.

[30] Saeed BQ, Al-Shahrabi R, Bolarinwa OA. Socio-demographic correlate of knowledge and practice toward COVID-19 among people living in Mosul-Iraq: A cross-sectional study. PloS one. 2021 Mar 31;16(3):e0249310. doi: 10.1371/journal.pone.0249310 33788835 PMC8011757

[31] Hussein NR, Naqid IA, Jacksi K, Abdi BA. Assessment of knowledge, attitudes, and practices toward COVID-19 virus among university students in Kurdistan region, Iraq: Online cross-sectional study. Journal of family medicine and primary care. 2020 Sep;9(9):4809. doi: 10.4103/jfmpc.jfmpc_870_20 33209805 PMC7652109

[32] Ahmad AR, Murad HR. The impact of social media on panic during the COVID-19 pandemic in Iraqi Kurdistan: online questionnaire study. Journal of medical Internet research. 2020 May 19;22(5):e19556. doi: 10.2196/19556 32369026 PMC7238863

[33] ODI. Women’s work: Mothers, children and the global childcare crisis. March 2016. Available from https://www.odi.org/sites/odi.org.uk/files/odi-assets/publications-opinion-files/10333.pdf [Accessed 06.06.2022].

[34] Dietrich & Simone E. Carter. (2017). Gender and Conflict Analysis in Isis Affected Communities of Iraq. Study produced by Oxfam with the support of UN Women in Iraq and the financial contribution of the Japanese Cooperation. Available from https://oxfamilibrary.openrepository.com/bitstream/handle/10546/620272/rr-gender-conflict-isis-affected-iraq-300517-en.pdf?sequence=1&isAllowed=y [Accessed on 03.07.22].

[35] Vilardo V. & Bittar S. (2018). Gender Profile in Iraq. Available from https://oxfamilibrary.openrepository.com/bitstream/handle/10546/620602/rr-gender-profile-iraq-131218-en.pdf;jsessionid=7818D86AE511FD0C917B2FC5DAA52BD3?sequence=1 [Access on 03.07.2022].

